# Predicting Cardiovascular Collapse in Critically Ill Patients During Intubation Induction: A Prospective Observational Study

**DOI:** 10.3390/medicina62010177

**Published:** 2026-01-15

**Authors:** Ömer Emgin, Gamze Taşkan, Aytuğ Yıldız, İmren Taşkıran, Engin Haftacı, Adnan Ata, Mehmet Yılmaz

**Affiliations:** Intensive Care Unit, Kocaeli City Hospital, University of Health Sciences, 41060 Izmit, Kocaeli, Turkey; gamztaskan@outlook.com (G.T.); aytugyildiz9093@gmail.com (A.Y.);

**Keywords:** peri-intubation cardiovascular collapse, shock indices, ketamine, propofol, intensive care unit

## Abstract

*Background and Objectives:* The study aimed to evaluate the predictive significance of Shock Indices and induction agents in predicting the risk of Peri-Intubation Cardiovascular Collapse (PIC) during intubation in the ICU. *Materials and Methods:* A total of 130 patients were analyzed in the study after dividing them into 2 groups based on the definition of PIC as Patients with PIC and Non-PIC Patients. PIC was defined as the detection of at least SBP < 65 mmHg measured at least once within 30 min after the intubation, SBP < 90 mmHg for 30 min, initiation of norepinephrine treatment, increasing the norepinephrine dose taken before the intubation, increasing SBP to >90 mmHg with >15 mL/kg crystalloid fluid infusion, or development of cardiac arrest. The relationship between Shock Index (SI), Diastolic Shock Index (DSI), Modified Shock Index (MSI), Age Shock Index (Age-SI), and induction agents (ketamine, propofol) and PIC was evaluated. *Results:* The PIC was detected in 62 patients (47.7%). Age-SI showed the highest predictive performance (AUC = 0.686, *p* < 0.001). Ketamine provided a protective effect (OR = 0.161, *p* = 0.003). Propofol (OR = 2.962, *p* = 0.048), age (OR = 1.065, *p* = 0.002), lactate (OR = 1.265, *p* = 0.047), and DSI (OR = 2.300, *p* = 0.037) were identified as independent risk factors. ICU mortality was significantly higher in the PIC group (74.2% vs. 20.6%, *p* < 0.001). *Conclusions:* Age, lactate, DSI, and Age-SI are valuable predictive parameters for PIC. Ketamine reduces the risk of PIC, while propofol increases it. These results support evidence-based risk assessment and induction agent selection in ICU intubation protocols.

## 1. Introduction

Endotracheal intubation is an essential component in airway management in critically ill patients in the Intensive Care Unit (ICU); however, it is also associated with serious complications such as Peri-Intubation Cardiovascular Collapse (PIC) [1]. Hemodynamic instability during intubation induction might increase morbidity and mortality [2]. Early diagnosis and prevention of PIC are critical to improve clinical outcomes [1,2]. Shock Indices such as Shock Index (SI), Diastolic Shock Index (DSI), Modified Shock Index (MSI), and the Age Shock Index (Age-SI) are simple prognostic parameters based on heart rate and blood pressure [3]. The normal range for SI is typically 0.5 to 0.7, with values greater than 0.9 signifying hemodynamic instability and an elevated risk of adverse clinical outcomes, such as increased mortality or ICU admission [3]. Similarly, MSI values exceeding 1.3 and higher DSI levels are indicative of greater cardiovascular stress and poorer prognosis in conditions like septic shock, often reflecting impaired diastolic function or vasodilatory states [4,5,6]. Age-SI, by multiplying age with SI, further refines risk assessment, where elevated scores correlate with heightened vulnerability to collapse, particularly in geriatric patients due to age-related physiological changes [4,5,6]. These parameters have been examined frequently as predictive parameters for conditions such as post-intubation hypotension in critically ill patients with a diagnosis of septic shock [4,5,6]. The DSI has also attracted attention in detecting early hemodynamic instability in sepsis patients. The Surviving Sepsis Campaign Guidelines indicate that DSI plays potential roles in the assessment of diastolic dysfunction [7]. There are insufficient studies evaluating the prediction of PIC during intubation induction by DSI and other Shock Indices [6].

The cardiovascular effects of sedative agents employed in intubation induction are an important factor in determining the risk of complications [8,9]. Ketamine supports hemodynamic stability with its sympathomimetic characteristics and is preferred in critically ill patients [9]. This effect occurs because ketamine stimulates catecholamine release and increases heart rate and blood pressure through β-adrenergic receptors [8]. In contrast, propofol is associated with dose-dependent hypotension and cardiovascular depression [8]. This effect of propofol can be explained by its inhibition of calcium channels in vascular smooth muscles, reducing vascular tone, and its negative inotropic effects [9,10]. However, the role of these agents in predicting PIC, together with Shock Indices, has not been investigated sufficiently. It has been reported that the creation and use of specific bundles will reduce peri-intubation risks [11].

This prospective observational study aims to evaluate the roles of DSI and other Shock Indices in predicting PIC during intubation induction. It also aims to provide evidence-based recommendations for sedative selection in the ICU by evaluating the cardiovascular effects of ketamine and propofol. The present study aimed to strengthen risk stratification to improve intubation safety in critically ill patients.

## 2. Materials and Methods

### 2.1. Study Design

This prospective, single-center, observational cohort study was conducted in Kocaeli City Hospital ICU between 15 July 2024, and 1 April 2025. The study aimed to identify the risk factors and predictive parameters of PIC during intubation induction in critically ill patients and was conducted in line with national and international ethical guidelines. The study protocol was approved by Kocaeli City Hospital Ethics Committee (approval code: 2024-49, approval date: 9 May 2024). Written informed consent was obtained from all participants or their legal representatives.

### 2.2. Patients

The study was initiated with 143 patients who were scheduled for intubation induction in the ICU. Based on the exclusion criteria, **8 patients who required emergency intubation because of cardiac arrest,** 3 patients who refused to participate in the study, and 2 patients under the age of 18 (a total of 13 patients) were excluded, and 130 patients were included in the analysis group. The sample size was calculated as a minimum of 102 patients, assuming an effect size of 0.5, an alpha margin of error of 0.05, and a power of 80%. The patients were divided into 2 groups as **Patients with PIC** (n = 62, 47.7%) and **Non-PIC Patients** (Non-PIC, n = 68, 52.3%). PIC was defined as SBP < 65 mmHg measured at least once within 30 min after the intubation, SBP < 90 mmHg for 30 min, initiation of norepinephrine treatment, increase in norepinephrine dose taken before intubation, increase in SBP to >90 mmHg with >15 mL/kg crystalloid fluid infusion, or development of cardiac arrest [12]. Demographic data, disease severity scores (Acute Physiology and Chronic Health Evaluation II [APACHE-II], Sequential Organ Failure Assessment [SOFA], Charlson Comorbidity Index [CCI]), duration of Invasive Mechanical Ventilation (IMV), length of ICU stay, and 30-day mortality were recorded along with the frequency and doses of induction agents (ketamine, propofol, midazolam, rocuronium), laboratory parameters on the day of intubation, vital signs and Shock Indices.

### 2.3. Data Collection

Laboratory parameters were recorded from the results obtained on the day of intubation. Vital signs (heart rate [HR], SBP, DBP, MBP) were recorded for 30 min before, during, and after the intubation. Invasive arterial monitoring was used in 90.8% (n = 118) of the patients, and non-invasive monitoring was used in 9.2% (n = 12) at 3 min intervals. The primary reasons for intubation were recorded and classified into five main categories: hypoxemic respiratory failure, hypercapnic respiratory failure, decreased level of consciousness/coma, hemodynamic instability/shock, and other. SI (HR/SBP), DSI (HR/DBP), MSI (HR/MBP), and Age-SI (age × SI) were calculated. The types and doses of induction agents and rocuronium use were listed. Hemoglobin, lactate, creatinine, Blood Urea Nitrogen (BUN), Alanine Aminotransferase (ALT), C-Reactive Protein (CRP), pH, and bicarbonate (HCO_3_) levels were analyzed from blood samples on the day of intubation. Receiver Operating Characteristic (ROC) analyses were performed to evaluate the predictive power of Shock Indices, and Area Under the Curve (AUC) values were reported.

### 2.4. Statistical Analysis

Continuous variables were expressed as median (Interquartile Range [IQR]) or Mean ± Standard Deviation (SD), and categorical variables as frequencies and percentages. Inter-group comparisons were made by the Mann–Whitney U-Test or the Independent Samples T-Test. Independent risk factors associated with PIC were determined by Multivariate Logistic Regression Analysis. Model fit was assessed by the Hosmer-Lemeshow Test, explanatory power was assessed by Nagelkerke R^2^. The predictive performance of SI, DSI, MSI, and Age-SI was reported with AUC values in ROC analyses. A separate multivariable logistic regression analysis was performed to identify independent predictors of 30-day mortality. Variables (PIC, APACHE-II score, ketamine use, propofol use, and Age-SI) were entered into the model. Age-SI was included instead of age to avoid multicollinearity. Model calibration was assessed using the Hosmer-Lemeshow test; explanatory power was reported with Nagelkerke R^2^. Results are presented as odds ratios with 95% confidence intervals.

Optimal threshold values, sensitivity, and specificity rates were also calculated, and statistical significance was accepted as *p* < 0.05. Analyses were performed with IBM SPSS Statistics (Version 26.0; IBM Corp., Armonk, NY, USA).

## 3. Results

**Figure 1:**
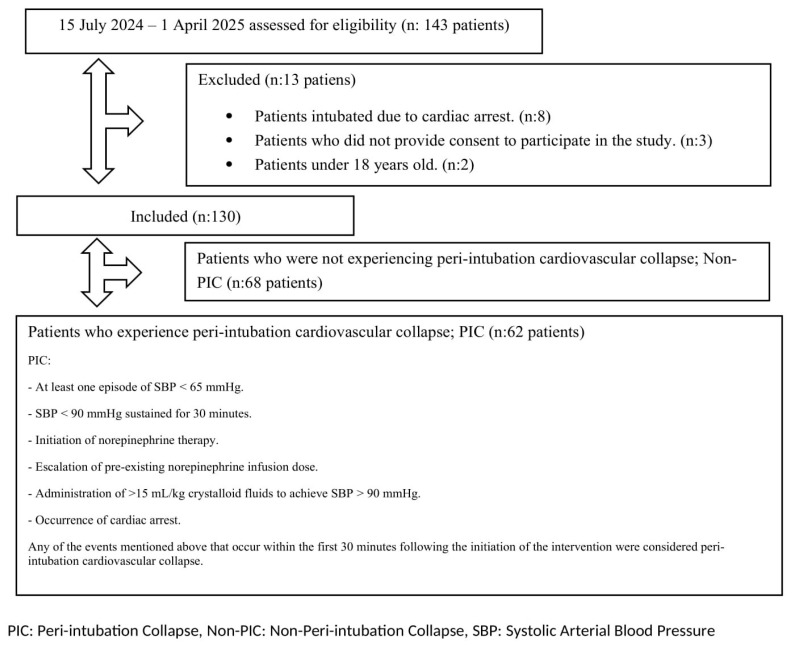
Flowchart of the study.

The study prospectively evaluated 143 patients scheduled for intubation induction in Kocaeli City Hospital ICU. After exclusion criteria (cardiac arrest [n = 8], refusal of consent [n = 3], under 18 years of age [n = 2]), 130 patients were analyzed. Participants were divided into 2 groups as PIC Patients (n = 62, 47.7%) and Non-PIC Patients (n = 68, 52.3%) (Figure 1). The mean age was 72.62 ± 13.43 years, and the age was significantly higher in the PIC group (76.55 ± 11.76 vs. 69.04 ± 13.94 years, *p* = 0.001). Sex distribution was similar (male: 56.2%, female: 43.8%; *p* = 0.773). Atrial Fibrillation had a higher prevalence in the PIC group (30.6% vs. 7.4%, *p* = 0.001). No significant differences were observed in APACHE-II, SOFA, or CCI scores between the groups. Similarly, ICU length of stay and duration of invasive mechanical ventilation were comparable, but 30-day mortality was significantly higher in the PIC group (Table 1).

Reasons for intubation were recorded and are presented in Table 2. The most common indications were decreased level of consciousness/coma (n = 94, 72.3%), hypoxemic respiratory failure (n = 77, 59.2%), hypercapnic respiratory failure (n = 55, 42.3%), and hemodynamic instability (n = 20, 15.4%). Multiple reasons were documented in 99 patients (76.2%). There were no significant differences in the distribution of intubation indications between the groups (Table 2).

Laboratory parameters on the day of intubation are given in Table 3. Lactate levels were found to be higher in the PIC group compared to the Non-PIC group. Hemoglobin, creatinine, CRP, and pH did not differ between the groups (Table 3).

In the comparison of induction agents employed for endotracheal intubation, significant differences were observed in the use of ketamine and propofol. Ketamine was administered less frequently in the PIC group compared to the Non-PIC group, while propofol was used more frequently in the PIC group. Ketamine and propofol doses did not differ significantly between the groups. Midazolam usage and doses were similar between the groups. Rocuronium was used in all patients, with no significant difference in doses between the groups. In direct comparison of ketamine and propofol, a significant relationship was found with PIC, with propofol used more frequently in the PIC group and ketamine more frequently in the Non-PIC group (Table 4).

Vital signs and Shock Indices are given in Table 5. Invasive monitoring was employed in most patients and was less common in the PIC group compared to the Non-PIC group, while non-invasive monitoring was more common in the PIC group. However, the difference between invasive and non-invasive monitoring was not statistically significant. Heart rate was significantly higher in the PIC group compared to the Non-PIC group. Systolic blood pressure, diastolic blood pressure, and mean blood pressure did not differ significantly between the groups. Shock Index, Diastolic Shock Index, and Modified Shock Index were significantly higher in the PIC group compared to the Non-PIC group. Similarly, Age-SI was significantly higher in the PIC group compared to the Non-PIC group (Table 5).

In the Multivariate Logistic Regression Analysis used to evaluate the risk factors for PIC, age, lactate, ketamine, propofol, and diastolic shock index (DSI) were found to be significant independent risk factors. Age significantly increased the risk of PIC (Odds Ratio [OR]: 1.065, 95% Confidence Interval [CI]: 1.024–1.107, *p* = 0.002), and lactate level was similarly associated with PIC (OR: 1.265, 95% CI: 1.003–1.596, *p* = 0.047). The use of ketamine significantly reduced the risk of PIC (OR: 0.161, 95% CI: 0.048–0.538, *p* = 0.003), whereas propofol use increased the risk of PIC (OR: 2.962, 95% CI: 1.010–8.685, *p* = 0.048). DSI showed a positive association with PIC (OR: 2.300, 95% CI: 1.050–5.040, *p* = 0.037). No significant relationship was detected between sex (OR: 0.860, 95% CI: 0.354–2.086, *p* = 0.738) and PIC, and the relationship between atrial fibrillation (OR: 3.415, 95% CI: 0.986–11.827, *p* = 0.053) and PIC was found to be borderline insignificant. The suitability of the model was confirmed by the Hosmer and Lemeshow test (χ^2^ = 6.065, df = 8, *p* = 0.640), and the Nagelkerke R^2^ value was calculated as 0.428, showing that the model has a significant explanatory power in explaining PIC (Table 6).

**Figure 2:**
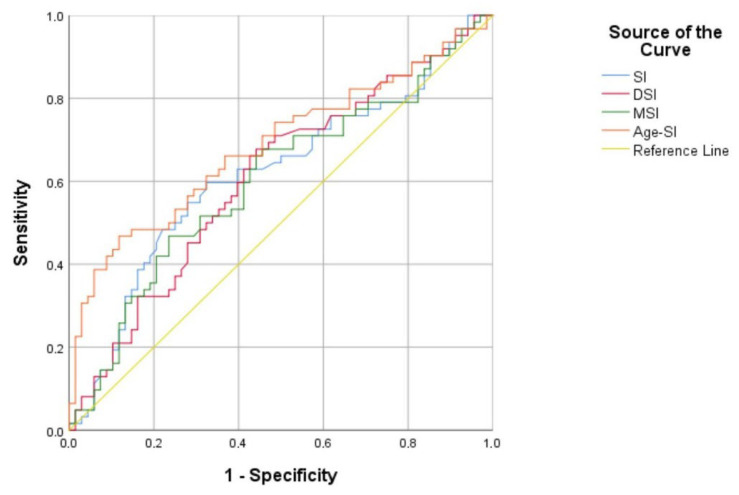
Receiver operating characteristic (ROC) curves to assess the ability of Shock Indices. Shock Index (SI), Diastolic Shock Index (DSI), Modified Shock Index (MSI), Age Shock Index (Age-SI), and Peri-intubation Collapse (PIC). The Area Under the Curve (AUC) values range from 0.603 (MSI) to 0.686 (Age-SI), with Age-SI demonstrating the highest discriminatory power (AUC = 0.686, 95% CI: 0.592–0.779, *p* < 0.001). All indices show statistically significant AUCs (*p* < 0.05), indicating moderate to good predictive performance, though Age-SI outperforms the others.

The predictive performance of Shock Indices for PIC was evaluated by ROC analysis. Age-SI showed the highest discrimination power (AUC: 0.686, 95% CI: 0.592–0.779, *p* < 0.001) and achieved 71.0% sensitivity and 54.4% specificity at a 59.42 cut-off point. SI achieved AUC 0.617 with 64.5% sensitivity and 51.5% specificity at 0.84 cut-off point (*p* = 0.019), while DSI achieved AUC 0.609 with 71.0% sensitivity and 51.5% specificity at 1.59 cut-off point (*p* = 0.029). MSI showed AUC 0.603 with 67.7% sensitivity and 54.4% specificity at a 1.26 cut-off point (*p* = 0.041). All indices exhibited statistically significant AUC values (*p* < 0.05), suggesting that they gave a moderate-to-good predictive performance, but the age-shock index outperformed the others. ROC curves visualized the ability of these indices to predict PIC (Figure 2, Table 7).

Multivariable logistic regression analysis identified independent predictors of 30-day mortality. The PIC was the strongest risk factor (OR 6.987, 95% CI 2.652–18.408, *p* < 0.001). Age-SI remained significant (OR 1.030, 95% CI 1.008–1.051, *p* = 0.006). APACHE-II score, propofol use, and ketamine use did not retain independent significance in the model. The model demonstrated excellent calibration (Hosmer-Lemeshow χ^2^ = 7.919, df = 8, *p* = 0.441) and substantial explanatory power (Nagelkerke R^2^ = 0.431) (Table 8).

## 4. Discussion

Although endotracheal intubation is a life-saving procedure for critically ill patients in the Intensive Care Unit, it is also associated with serious complications such as peri-intubation cardiovascular collapse [1,12]. In the present study, the PIC rate was found to be 47.7%, which is similar to the 43.4% rate reported in the INTUBE study [12]. This rate highlights the hemodynamic risks of intubation in critically ill patients [4,11]. The study systematically evaluated the roles of age, sex, atrial fibrillation, lactate level, DSI, and the induction agents ketamine and propofol in predicting PIC. The results of the present study showed that age, lactate, DSI, ketamine, and propofol were statistically significantly and independently associated with PIC.

Age-SI showed the highest predictive performance for PIC prediction among Shock Indices. Integration of the age factor is pathophysiologically consistent with the tendency for decreased cardiovascular reserve and increased hemodynamic instability in elderly patients [2,13]. Vascular stiffness with aging is associated with collagen deposition and elastin loss, leading to decreased arterial compliance and impaired baroreceptor sensitivity [14]. These mechanisms increase sensitivity to increased hemodynamic stress during intubation [15]. The INTUBE study also identified age as an independent risk factor for PIC [12]. The high prevalence of cardiovascular comorbidities in elderly patients may also increase the risk of PIC [16]. Age-SI parameter, which is evaluated together with age and SI and has a statistically significant relationship with PIC, might be more predictive for PIC.

The clinical application of Age-SI in risk classification protocols for the PIC group offers a practical, bedside tool to enhance patient safety during intubation in the ICU. Age-SI is calculated simply as age (in years) multiplied by the Shock Index, using pre-intubation vital signs, which are routinely available without additional equipment or laboratory tests. Based on our ROC analysis, a cut-off value of 59.42 provides moderate sensitivity (71.0%) and specificity (54.4%), allowing clinicians to stratify patients into low- and high-risk categories. For instance, in patients with Age-SI >59.42, heightened vigilance is warranted, prompting preemptive interventions such as fluid optimization (e.g., >15 mL/kg crystalloid bolus if hypovolemia is suspected), vasopressor standby (e.g., norepinephrine initiation or dose escalation), or selection of hemodynamically stable induction agents like ketamine over propofol, as supported by our findings (OR = 0.161 for ketamine’s protective effect).

DSI might be an independent predictor of PIC by reflecting diastolic dysfunction and loss of vascular tone [17]. Increased DSI is associated with decreased systemic vascular resistance and diastolic dysfunction, which can be explained by molecular mechanisms such as endothelial dysfunction and nitric oxide overproduction [18]. Decreased systemic vascular resistance in sepsis and hypoperfusion increases DSI, indicating early hemodynamic instability [17]. The association of low diastolic blood pressure with PIC is also supported in the literature [17]. The combined use of Age-SI, which has the highest predictive value, and DSI, which has the highest sensitivity, will be more valuable for PIC prediction.

Shock indices, calculated by integrating heart rate with blood pressure, are more sensitive in detecting hemodynamic instability. Blood pressure may mask early collapse risk due to compensatory mechanisms, while shock indices better reflect cardiovascular stress [19].

Ketamine reduces the risk of PIC with its sympathomimetic effects, while propofol increases the risk with its hypotensive effects. Ketamine increases catecholamine release over N-Methyl-D-Aspartate (NMDA) receptor antagonism and supports heart rate and blood pressure over β-adrenergic stimulation [20], which preserves cardiovascular stability, especially in hypovolemic or septic shock [21]. In contrast, propofol reduces vascular tone and suppresses myocardial contractility by inhibiting calcium channels in vascular smooth muscle cells [22]. Also, propofol has been shown to reduce baroreflex sensitivity, which increases the risk of hypotension [21,22]. The INTUBE study confirmed the association of propofol with PIC [12]. The results of the present study, in line with the literature data, support the use of ketamine and recommend that propofol be selected carefully [23,24,25]. In this study, the fact that the doses were similar between the groups suggests that the effects depend on pharmacodynamic characteristics. It can also be interpreted as a result of the ketamine and propofol doses remaining in the therapeutic range in both groups. As stated in the Surviving Sepsis Campaign guide, when the data of this study are taken into consideration, Ketamine appears to be the agent of choice to prevent hypotension and other adverse effects [9].

Reasons for intubation were similar between the PIC and non-PIC groups (Table 2), with decreased level of consciousness/coma and hypoxemic respiratory failure being the most common indications and multiple indications present in 76.2% of patients. In the large multicenter INTUBE study, reason for intubation was an independent predictor of peri-intubation cardiovascular collapse [12]. In contrast, no significant univariate association was observed between any intubation indication and PIC in the present single-centre cohort. The strong protective effect of ketamine and the independent risk associated with propofol thus persist independently of the primary reason for intubation.

When comorbidities were evaluated in the study, Atrial Fibrillation was found to be a significant risk factor for PIC in the univariate analysis. This significance was limited in the multivariate analysis. It would be useful to keep in mind that AF can cause irregular ventricular filling and decreased cardiac output, leading to hemodynamic instability in patients undergoing intubation [26,27,28]. The results revealed the need for larger studies to evaluate the relationship between atrial fibrillation and PIC. The increase in lactate is associated with the increase in lactate dehydrogenase activity, which catalyzes the conversion of pyruvate to lactate under hypoxic conditions. The high lactate in the PIC group confirms the prognostic value of hypoperfusion [28,29,30].

Although general intensive care mortality rates are around 25–35%, this rate is higher in malignancy, sepsis/septic shock, and geriatric patients [31,32,33]. Old age and malignancy increase the susceptibility to hemodynamic instability and poor clinical outcomes due to systemic inflammation and decreased physiological reserve [34]. Malignancy may increase the risk of mortality through increased proinflammatory cytokines (e.g., IL-6, TNF-α) in the tumor microenvironment, decreased catecholamine reserve, and many other mechanisms [35]. The relatively high mortality rate in the study may be explained by the elderly population and high prevalence of malignancy in this cohort.

The PIC emerged as by far the strongest independent predictor of 30-day mortality (OR ≈ 7), consistent with the INTUBE study and confirming that haemodynamic decompensation during intubation represents a pivotal prognostic event beyond baseline illness severity [12]. Age-SI was the only other parameter that retained independent prognostic value, reinforcing Age-SI as a simple yet powerful composite marker that integrates age-related physiological vulnerability with pre-intubation haemodynamic stress, outperforming raw age and APACHE-II in this context [13,14,15]. Although APACHE-II score and induction agent choice (ketamine vs. propofol) were significant in univariate analysis, they lost independent significance after adjustment for PIC and Age-SI, most likely because their prognostic influence is largely mediated or overpowered by these two dominant predictors. These findings suggest that systematic efforts to prevent PIC and routine pre-intubation calculation of Age-SI could offer practical and effective means to improve risk stratification and potentially enhance outcomes in critically ill patients requiring emergency intubation.

This prospective analysis adds a direct comparison of shock indices for peri-intubation risk prediction and further supports the independent association of cardiovascular collapse with short-term mortality while favouring ketamine use in higher-risk patients.

The strengths of the study include the prospective design, standardized data collection protocol, and high invasive monitoring rate (90.8%). The high explanatory power of Multivariate Logistic Regression Analysis (Nagelkerke R^2^ = 0.428) and the verification of model fit by the Hosmer-Lemeshow Test (χ^2^ = 6.065, *p* = 0.640) increased the reliability of the results. The main limitations of this single-center study are its moderate sample size and limited generalizability. Exact pre-induction FiO_2_ values were not systematically recorded, preventing reliable calculation of the SpO_2_/FiO_2_ ratio—an independent predictor in the INTUBE study [12]. Echocardiographic assessment of left ventricular systolic and diastolic function was available in only a minority of patients, and detailed pre-intubation vasopressor requirements and post-intubation ventilator settings (e.g., PEEP levels) were not uniformly documented. Finally, because of the observational design, induction agent selection was at the discretion of the treating clinician, which may introduce confounding by indication despite multivariate adjustment. Randomized controlled trials and larger multicenter cohorts incorporating these parameters and machine learning–based risk models are needed to validate our findings and develop robust clinical prediction tools [36].

## 5. Conclusions

The results of the present study showed that PIC is a serious complication detected at a high rate during intubation induction in critically ill patients and is associated with mortality. Shock Indices were found to be significant parameters in the prediction of PIC. Routine use of Age-SI, the shock index with the highest predictive value for PIC, and DSI, which is independently associated with PIC, in clinical practice has the potential to reduce intubation-related complications. Ketamine reduced the risk of PIC, while propofol increased it. Age, lactate, DSI, and the agents selected at induction (ketamine and propofol) are independently associated parameters with PIC. These findings support the adoption of evidence-based approaches in pre-intubation risk stratification and induction agent selection in the ICU. Future multicenter studies should evaluate the validation of these parameters and their integration into clinical decision support systems.

## Figures and Tables

**Table 1:** Comparison of baseline demographic data/scores between PIC and Non-PIC Groups.

Baseline Variables	All Patients n:130 (100%)	PIC n:62 (47.7%)	Non-PIC n:68 (52.3%)	*p*-Value
Age (mean years)	72.62 ± 13.43	76.55 ± 11.76	69.04 ± 13.94	**0.001**
Sex (n (%))				
Female	57 (43.8%)	28 (45.2%)	29 (42.6%)	
Male	73 (56.2%)	34 (54.8%)	39 (57.4%)	0.773
Comorbidities (n (%))				
Hypertension	63 (48.8%)	34 (54.8%)	29 (42.6%)	0.165
Diabetes Mellitus	53 (40.8%)	20 (32.3%)	33 (48.5%)	0.059
Malignancy	44 (33.8%)	21 (33.9%)	23 (33.8%)	0.995
Coronary Artery Disease	30 (23.1%)	16 (25.8%)	14 (20.6%)	0.481
Congestive Heart Disease	25 (19.2%)	12 (19.4%)	13 (19.1%)	0.971
Atrial Fibrillation	24 (18.5%)	19 (30.6%)	5 (7.4%)	**0.001**
Chronic Kidney Disease	24 (18.5%)	10 (16.1%)	14 (20.6%)	0.513
Severity Scores				
APACHE-II scores	31.40 ± 10.13	32.76 ± 10.59	30.18 ± 9.61	0.148
SOFA scores	10.50 (6.75–13.00)	10.50 (8.00–13.00)	10.50 (5.25–13.00)	0.662
CCI	6.00 (5.00–8.00)	6.00 (5.00–8.00)	6.00 (4.00–7.00)	0.064
Duration of IMV (days)	10.00 (3.00–25.00)	8 (3.00–18.25)	12.50 (3.00–30.00)	0.099
Length of stay ICU (days)	15.50 (7.00–30.25)	13.50 (7.00–26.25)	21.00 (7.00–38.00)	0.128
Mortality 30-day (n (%))	60 (46.2%)	46 (74.2%)	14 (20.6%)	**<0.001**

PIC: Peri-intubation Collapse, Non-PIC: Non-Peri-intubation Collapse, IMV: Invasive Mechanical Ventilation, APACHE-II: Acute Physiologic and Chronic Health Evaluation-II, SOFA: Sequential Organ Failure Assessment, CCI: Charlson Comorbidity Index, ICU: Intensive Care Unit. Bold formatting denotes statistical significance at *p* < 0.05.

**Table 2:** Reasons for Intubation.

Reason	PIC n:62 (47.7%)	Non-PIC n:68 (52.3%)	*p*-value
Hypoxemic respiratory failure	37 (59.7%)	40 (58.8%)	1.000
Hypercapnic respiratory failure	23 (37.1%)	32 (47.1%)	0.332
Decreased level of consciousness/coma	46 (74.2%)	48 (70.6%)	0.793
Hemodynamic instability/shock	13 (21.0%)	7 (10.3%)	0.149
Other	4 (6.5%)	3 (4.4%)	0.900

PIC: Peri-intubation Collapse, Non-PIC: Non-Peri-intubation Collapse (Patients could have more than one indication for intubation; percentages are calculated within each group and therefore sum to more than 100%).

**Table 3:** Comparison of laboratory data on the day of intubation between PIC and Non-PIC Groups.

Laboratory Data	All Patients n:130 (100%)	PIC n:62 (47.7%)	Non-PIC n:68 (52.3%)	*p*-Value
Hemoglobin (g/dL)	9.70 (8.37–11.32)	9.65 (8.00–10.85)	9.80 (8.50–11.87)	0.123
Leukocyte (10^3^/μL)	11.25 (7.80–17.16)	11.39 (7.09–17.76)	11.24 (8.32–16.72)	0.638
Platelet (10 ^3^/μL)	180.00 (94.50–280.25)	168.50 (95.00–286.25)	181.50 (93.50–268.50)	0.789
Glucose (mg/dL)	136.50 (111.75–178.50)	141.00 (113.75–197.25)	135.00 (101.75–177.75)	0.397
Albumin (g/L)	25.00 (22.00–32.00)	25.00 (21.00–32.25)	26.50 (23.00–31.00)	0.455
Creatinine (mg/dL)	0.90 (0.59–2.02)	0.94 (0.60–2.07)	0.85 (0.50–2.06)	0.549
BUN (mg/dL)	33.00 (21.00–49.25)	34.00 (21.75–45.25)	31.50 (18.00–53.75)	0.695
ALT (U/L)	21.00 (11.00–39.25)	23.00 (10.00–41.25)	20.50 (11.25–37.50)	0.902
CRP (mg/L)	113.00 (66.75–200.25)	125.50 (71.00–187.25)	106.00 (56.87–205.25)	0.801
pH	7.33 (7.23–7.43)	7.31 (7.23–7.42)	7.35 (7.23–7.44)	0.461
HCO_3_ (mmol/L)	24.95 (20.30–31.00)	23.00 (20.00–30.00)	25.75 (21.10–32.55)	0.139
Lactate (mmol/L)	1.69 (1.20–3.13)	2.07 (1.39–3.42)	1.43 (1.07–2.82)	**0.008**

PIC: Peri-intubation Collapse, Non-PIC: Non-Peri-intubation Collapse, BUN: Blood Urea Nitrogen, ALT: Alanine Amino-Transferase, CRP: C-Reactive Protein, HCO_3_: Bicarbonate. Bold formatting denotes statistical significance at *p* < 0.05.

**Table 4:** Comparison of induction agents used for intubation between PIC and Non-PIC Groups.

Induction Agents	All Patients n: 130 (100%)	PIC n:62 (47.7%)	Non-PIC n:68 (52.3%)	*p*-Value
Ketamine (n (%))	43 (33.1%)	10 (16.1%)	33 (48.5%)	**<0.001**
Ketamine dose (mg/kg)	1.28 (0.90–1.52)	1.20 (1.03–1.33)	1.29 (0.76–1.57)	0.989
Propofol (n (%))	52 (40%)	32 (51.6%)	20 (29.4%)	**0.010**
Propofol dose (mg/kg)	0.82 (0.69–1.17)	0.80 (0.68–1.10)	0.93 (0.73–1.26)	0.429
Midazolam (n (%))	35 (26.9%)	20 (33.2%)	15 (22.1%)	0.190
Midazolam dose (mg/kg)	0.02 (0.02–0.04)	0.02 (0.02–0.03)	0.02 (0.01–0.04)	0.488
Rocuronium (n (%))	130 (100%)	62 (100%)	68 (100%)	-
Rocuronium dose (mg/kg)	0.62 (0.49–0.68)	0.57 (0.43–0.67)	0.63 (0.52–0.70)	0.102
Ketamine Versus Propofol				**<0.001**
Ketamine (n (%))	43 (33.1%)	10 (16.1%)	33 (48.5%)	
Propofol (n (%))	52 (40%)	32 (51.6%)	20 (29.4%)	

PIC: Peri-intubation Collapse, Non-PIC: Non-Peri-intubation Collapse. Bold formatting denotes statistical significance at *p* < 0.05.

**Table 5:** Comparison of vital signs and Shock Indices between PIC and Non-PIC groups.

	All Patients n: 130 (100%)	PIC n:62 (47.7%)	Non-PIC n:68 (52.3%)	*p*-Value
Invasive versus Non-Invasive				0.068
Invasive monitoring	118 (90.8%)	53 (85.5%)	65 (95.6%)	
Non-invasive monitoring	12 (9.2%)	9 (14.5%)	3 (4.4%)	
HR (bpm)	106.67 (88.00–123.50)	114.50 (98.00–130.00)	97.50 (83.40–111.50)	**<0.001**
SBP (mmHg)	119.00 (91.25–135.00)	128.00 (91.67–139.00)	116.00 (102.00–132.55)	0.451
DBP (mmHg)	60.44 (50.92–70.00)	60.20 (50.88–71.00)	60.75 (51.00–69.25)	0.478
MBP (mmHg)	80.50 (66.67–91.00)	82.50 (66.33–95.25)	79.00 (67.00–88.50)	0.271
SI	0.86 (0.67–1.13)	0.97 (0.75–1.28)	0.84 (0.69–0.97)	**0.021**
DSI	1.72 (1.35–2.19)	1.86 (1.47–2.33)	1.58 (1.30–2.06)	**0.033**
MSI	1.34 (1.05–1.61)	1.40 (1.10–1.84)	1.21 (1.03–1.49)	**0.043**
Age-SI	63.26 (50.14–80.99)	70.13 (58.35–101.25)	58.54 (47.27–69.39)	**<0.001**

PIC: Peri-intubation Collapse, Non-PIC: Non-Peri-intubation Collapse, HR: Heart Rate, SBP: Systolic Blood Pressure, DBP: Diastolic Blood Pressure, MBP: Mean Arterial Pressure, SI: Shock Index, DSI: Diastolic Shock Index, MSI: Modified Shock Index, Age-SI: Age Shock Index. Bold formatting denotes statistical significance at *p* < 0.05.

**Table 6:** Multivariate logistic regression analysis for risk factors for Peri-intubation Collapse.

Risk Factors	Odds Ratio	95% Confidence Interval	*p* Value
Age (year)	1065	1024–1107	**0.002**
Sex (n (%))	0.860	0.354–2.086	0.738
Atrial Fibrillation	3415	0.986–11.827	0.053
Lactate (mmol/L)	1265	1003–1596	**0.047**
Ketamine (n (%)	0.161	0.048–0.538	**0.003**
Propofol (n (%))	2962	1010–8685	**0.048**
Diastolic Shock Index	2300	1050–5040	**0.037**

The Hosmer and Lemeshow Test (χ^2^ = 6.065, df = 8, *p* = 0.640) indicates a satisfactory model fit. The Nagelkerke R^2^ of 0.428 indicates a substantial explanatory power for peri-intubation collapse. Bold formatting denotes statistical significance at *p* < 0.05.

**Table 7:** Predictive Performance and ROC Analysis Results of Shock Indices for Peri-intubation Collapse.

	Cut off	Sensitivity	Specificity	AUC	*p* Value
SI	0.84	64.5%	51.5%	0.617	**0.019**
DSI	1.59	71.0%	51.5%	0.609	**0.029**
MSI	1.26	67.7%	54.4%	0.603	**0.041**
Age-SI	59.42	71.0%	54.4%	0.686	**<0.001**

AUC: Area Under the Curve, SI: Shock Index, DSI: Diastolic Shock Index, MSI: Modified Shock Index, Age-SI: Age Shock Index. Bold formatting denotes statistical significance at *p* < 0.05.

**Table 8:** Multivariate Logistic Regression Analysis for Risk Factors for 30-Day Mortality.

Risk Factors	Odds Ratio	95% Confidence Interval	*p* Value
PIC	6.987	2.652–18.408	**<0.001**
APACHE II	1.019	0.976–1.065	0.394
Propofol (n (%))	1.191	0.400–3.545	0.754
Ketamine (n (%))	0.793	0.235–2.678	0.709
Age-SI	1.030	1.008–1.051	**0.006**

PIC: Peri-intubation Collapse, APACHE-II: Acute Physiologic and Chronic Health Evaluation-II, Age-SI: Age Shock Index. The Hosmer-Lemeshow goodness-of-fit test confirmed excellent model calibration (χ^2^ = 7.919, df = 8, *p* = 0.441). The Nagelkerke R^2^ of 0.431 indicates substantial explanatory power of the model for 30-day mortality. Bold formatting denotes statistical significance at *p* < 0.05.

## Data Availability

Data is available upon request to the corresponding author. It is not publicly available due to confidentiality reasons.
